# Risk factors for pancreatitis occurrence after gallstone treatment using endoscopic retrograde cholangiopancreatography

**DOI:** 10.4314/ahs.v23i2.26

**Published:** 2023-06

**Authors:** Feibao Jiang, Jilong Zhang, Zhenghua Hu

**Affiliations:** Surgical Department Ward One, People's Hospital of Changshan City, Changshan 324200, Zhejiang Province, China

**Keywords:** risk factor, endoscopic retrograde cholangiopancreatography, pancreatitis, gallstone, pancreatic duct stenting

## Abstract

**Background:**

Patients with gallstones are prone to pancreatitis after treatment using endoscopic retrograde cholangiopancreatography (ERCP). The aim of this study was to explore the risk factors for pancreatitis occurrence after gallstone treatment using ERCP.

**Methods:**

A total of 193 patients treated from October 2017 to October 2020 were assigned into pancreatitis group (n=55) and non-pancreatitis group (n=138). Multivariate logistic regression analysis was utilized to analyse the risk factors for post-ERCP pancreatitis. The discrimination and accuracy of an established nomogram model were evaluated using receiver operating characteristic and calibration curves, respectively.

**Results:**

The incidence rate of pancreatitis was 28.50% (55/193). Young age, long course of disease, gallbladder wall thickness >3 mm, sand-like stones, history of pancreatic disease, number of intubation ≥2 and absence of pancreatic duct stenting were risk factors for post-ERCP pancreatitis (P<0.05). The established model had high discrimination and accuracy. The incidence rates of pancreatitis in patients with and without pancreatic duct stenting were 11.84% (9/76) and 39.31% (46/117), respectively. The patients undergoing pancreatic duct stenting had lower serum amylase levels 6, 12 and 24 h after ERCP than those of patients who did not.

**Conclusion:**

Patients with gallstones have a higher risk of developing pancreatitis. Young age, long course of disease, gallbladder wall thickness >3 mm, sand-like stones, history of pancreatic disease, pancreatic duct visualization and number of intubation ≥2 are risk factors for post-ERCP pancreatitis.

## Introduction

Gallstones have a mounting incidence rate in recent years, which mostly occur in middle-aged people and may induce abdominal pain, seriously threatening human health 1,2. At present, gallstones are mainly treated by surgery 3, and endoscopic retrograde cholangiopancreatography (ERCP) has been widely applied in diagnosing biliary system diseases^4^,^5^. Besides, gallstones can be effectively treated by ERCP, with rapid postoperative recovery^6^. Nonetheless, the risk of post-ERCP complications, especially pancreatitis (in most cases), still exists^7^. Nowadays, it is well-documented that pancreatic duct stenting not only exerts a preventive effect on pancreatitis^8^, but also relieves refractory pain resulting from pancreatic duct stones^9^. However, it is difficult to treat post-ERCP complications in patients with gallstones, with long treatment course and poor prognosis^10^,^11^. Based on these, identifying related risk factors contributes to preventing the occurrence of complications such as pancreatitis.

Hence, we herein investigated the risk factors for post-ERCP pancreatitis in patients with gallstones and assessed the preventive effect of pancreatic duct stenting, aiming to providing valuable guidance for effective prevention after operation.

## Materials and Methods

### Subjects

A total of 193 patients with gallstones treated in our hospital from October 2017 to October 2020 were included in this study. These patients were assigned to pancreatitis group (n=55) and non-pancreatitis group (n=138) according to the presence or absence of post-ERCP pancreatitis. In the pancreatitis group, there were 23 males and 32 females aged 49-80 years old, with an average of (55.61±6.67) years old. In the non-pancreatitis group, there were 49 males and 89 females aged 48-80 years old, averagely (53.73±7.09) years old. The two groups had comparable baseline data (P>0.05). Based on whether pancreatic duct stenting was performed or not, the patients were divided into surgery group (n=76) and non-surgery group (n=117). This study was approved by the ethics committee of the hospital, and informed consent was obtained from the patients and their family members.

### Inclusion and exclusion criteria

Inclusion criteria were as follows: a) patients diagnosed as gallstones by abdominal ultrasonography, b) those who met the diagnostic criteria for gallstones in accordance with the Consensus on diagnosis and treatment of chronic cholecystitis and gallstones in China, c) those with a stone diameter of <3 cm, d) those who underwent ERCP in our hospital for the first time, and e) those ≥48 years old.

Exclusion criteria involved: a) patients with obstructive or acute cholecystitis, b) those with a large diameter of stones requiring stent placement, c) those with stones at other sites, d) those with chronic or acute pancreatitis, e) those taking nonsteroidal anti-inflammatory drugs, f) those with severe heart and renal diseases, or g) those who were pregnant.

### Surgical methods

The procedure of ERCP was detailed as follows: Intravenous anesthesia was performed, and the patients were given electrocardiogram and oxygen saturation monitoring throughout ERCP. The electronic duodenoscope was inserted into the descending portion of the duodenum, and different stone extraction regimens were utilized depending on the stone conditions.

The procedure of pancreatic duct stenting was described as follows: After entering the abdominal cavity, the gastrocolic ligament was first explored and incised. The common hepatic artery and gastroduodenal artery were dissected, and the space between the superior mesenteric vein and pancreatic neck was separated. A 5F ureteral catheter was inserted into the duodenum through the cut of pancreatic duct, and then the catheter, through intraoperative gastroscopy, was confirmed to be positioned well. Next, the pancreatic ducts at both ends of the cut were sutured and closed to complete pancreatic duct stenting and repair. After confirming that there was no active bleeding at the surgical wound, an abdominal drainage tube was retained next to the pancreatic wound^12^.

### Observation indicators

Age, course of disease, gallbladder wall thickness, stone appearance, number of intubations, history of pancreatic disease, gender, smoking history, drinking history, body mass index, marital status, annual family income, education level, gallbladder size, living environment, operation time, stone diameter, number of stones, number of angiography and serum amylase levels 6, 12 and 24 h after ERCP were recorded.

### Diagnostic criteria for post-ERCP pancreatitis

The individuals who met at least two of the following criteria were diagnosed as pancreatitis13: a) patients who presented with acute, sudden, persistent and severe epigastric pain with the potential to radiate to the back 24 h after operation, b) those with serum amylase and/or lipase activity ≥3 upper limit of normal value (ULN), or c) those with pancreatic edema or peripancreatic effusion displayed on abdominal enhanced CT or MRI images.

### Statistical analysis

SPSS 22.0 software was employed for statistical analysis. Measurement data were expressed as mean ± standard deviation (-χ±s) and compared using the t-test. Count data were expressed as percentage and compared using the χ^2^ test. Multivariate logistic regression analysis was used to analyse the risk factors. R software was applied to construct a nomogram model. Besides, the discrimination and accuracy of the model were evaluated using the receiver operating characteristic (ROC) curve and calibration curve, respectively. P<0.05 was considered statistically significant.

## Results

### Occurrence of pancreatitis

Among the 193 patients with gallstones, there were 72 males and 121 females aged 48-80 years old, averagely (54.13±6.87) years old. The course of disease was 2-11 years, with an average of (6.52±0.87) years. Additionally, 116 patients had 1-3 gallstones and 77 patients had >3 gallstones. At 24 h after ERCP, the incidence rate of pancreatitis in patients with gallstones was 28.50% (55/193).

### Univariate analysis results

The results of univariate analysis manifested that the differences in age, course of disease, gallbladder wall thickness, stone appearance, number of intubation, history of pancreatic disease and pancreatic duct stenting were statistically significant between the two groups (P<0.05). Furthermore, the patients who developed pancreatitis were younger and had a longer course of disease, a greater number of intubations, gallbladder wall thickness >3 mm, sand-like stones, and history of pancreatic disease in most cases. In addition, no significant differences were observed in difficult cannulation, biliary sphincterotomy, ERCP procedure time, gender or smoking history between the two groups (P>0.05) (Table 1).

**Table 1:** Univariate analysis results

Variable	Pancreatitis group (n=55)	Non-pancreatitis group (n=138)	*t*/χ^2^	P
Age	55.61±6.67	53.73±7.09	1.691	0.046
Gender			0.670	0.413
Male	23 (41.82)	49 (35.51)		
Female	32 (58.18)	89 (64.49)		
Smoking history			1.435	0.231
Yes	16 (29.09)	29 (21.01)		
No	39 (70.91)	109 (78.99)		
Drinking history			0.634	0.426
Yes	20 (36.36)	42 (30.43)		
No	35 (63.64)	96 (69.57)		
Body mass index			0.005	0.945
≥25 kg/m^2^	26 (47.27)	66 (47.83)		
<25 kg/m^2^	29 (52.73)	72 (52.17)		
Marital status			0.027	0.870
Unmarried, divorced or widowed	4 (7.27)	11 (7.97)		
Married	51 (92.73)	127 (92.03)		
Annual family income			0.001	0.990
≥50,000 RMB	12 (21.82)	30 (21.74)		
<50,000 RMB	43 (78.18)	108 (78.26)		
Education level			0.122	0.726
Senior high school and above	22 (40.00)	59 (42.75)		
Senior high school below	33 (60.00)	79 (57.25)		
Course of disease	7.50±1.36	6.91±1.25	3.962	0.000
Gallbladder size			0.150	0.699
Normal	26 (47.27)	61 (44.20)		
Abnormal	29 (52.73)	77 (55.80)		
Living environment			1.458	0.227
Urban area	30 (54.55)	62 (44.93)		
Rural area	25 (45.45)	76 (55.07)		
Operation time (min)	42.36±2.59	42.37±2.41	0.025	0.490
Gallbladder wall thickness			8.606	0.003
>3 mm	21 (38.18)	23 (16.67)		
≤3 mm	34 (61.82)	105 (76.09)		
Stone diameter			0.063	0.802
≤1 cm	26 (47.27)	68 (49.28)		
>1 cm	29 (52.73)	70 (50.72)		
Number of stones			0.400	0.527
1-3	35 (63.64)	81 (58.70)		
>3	20 (36.36)	57 (41.30)		
Stone appearance			17.064	0.000
Sand-like	34 (61.82)	41 (29.71)		
Granular	21 (38.18)	97 (70.29)		
Number of angiography (times)	2.36±0.14	2.25±0.39	2.037	0.021
Number of intubation (times)	3.19±0.57	1.84±0.37	19.420	0.000
History of pancreatic disease			7.412	0.006
Yes	43 (78.18)	79 (57.25)		
No	12 (21.82)	59 (42.75)		
Pancreatic duct stenting			17.067	0.000
Yes	9	67		
No	46	71		
Difficult cannulation	21 (38.18)	55 (39.86)	0.046	0.830
Biliary sphincterotomy	18 (32.73)	50 (3.23)	0.212	0.645
ERCP procedure time	21.26±3.42	20.34±3.56	1.639	0.103

### Multivariate logistic regression analysis results

Multivariate logistic regression analysis was conducted with post-ERCP pancreatitis in patients with gallstones as the dependent variable (occurrence=1, no occurrence=0) and indicators with statistical significance (P<0.05) as the independent variables, which were then assigned (Table 2). The results revealed that young age, long course of disease, gallbladder wall thickness >3 mm, sand-like stones, history of pancreatic disease, number of intubation ≥2 and absence of pancreatic duct stenting were risk factors for post-ERCP pancreatitis in patients with gallstones (P<0.05) (Table 3).

**Table 2:** Variable assignment table

Independent variable	Category	Value assignment
Age	Continuous variable	Measured value
Course of disease	Continuous variable	Measured value
Gallbladder wall thickness >3 mm	Dichotomous variable	Yes=1, no=0
Sand-like stones	Dichotomous variable	Yes=1, no=0
Number of intubation ≥2	Dichotomous variable	Yes=1, no=0
History of pancreatic disease	Dichotomous variable	Yes=1, no=0
Absence of pancreatic duct stenting	Dichotomous variable	Yes=1, no=0

**Table 3:** Multivariate logistic regression analysis results

Variable	Regression coefficient	Standard error	Wald χ^2^	P	OR	95% CI
Young age	0.594	0.068	76.416	0.001	1.812	1.714~2.961
Course of disease	0.538	0.149	13.021	0.001	1.712	1.631~2.912
Gallbladder wall thickness >3 mm	0.212	0.103	4.232	<0.001	1.236	1.023~2.947
Sand-like stones	0.224	0.115	3.792	0.002	1.251	1.147~2.852
Number of intubation ≥2	0.487	0.167	8.516	0.001	1.628	1.364~2.997
History of pancreatic disease	0.226	0.108	4.392	<0.001	1.254	1.039~2.847
Absence of pancreatic duct stenting	0.491	0.158	9.658	<0.001	1.634	1.236~2.062

### Nomogram model

**Figure 1:**
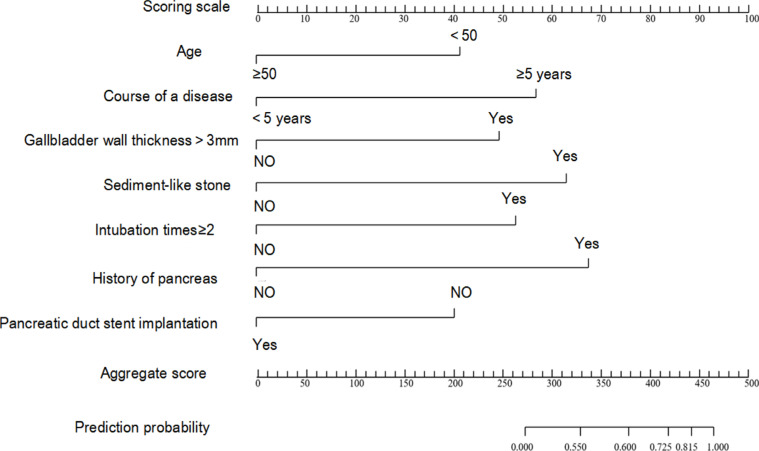
Nomogram model

The independent risk factors identified from multivariate analysis were utilized to construct a nomogram prediction model for post-ERCP pancreatitis in patients with gallstones, thereby predicting the probability of post-ERCP pancreatitis (Figure 1). First, the value for each variable was positioned on the item scale, and a vertical line was drawn on the first row of the single-item score axis to correspond to the value point of each variable, giving the score of each variable. Then the sum of the individual scores for all variables corresponded vertically to the probability of occurrence. The results indicated that young age, long course of disease, gallbladder wall thickness >3 mm, sand-like stones, history of pancreatic disease, pancreatic duct visualization, number of intubation ≥2 and absence of pancreatic duct stenting were totally scored 441 points. The risk value for post-ERCP pancreatitis in patients with gallstones was 0.815, that is, the predicted probability for post-ERCP pancreatitis in patients with gallstones was 81.5%.

### Model validation

**Figure 2:**
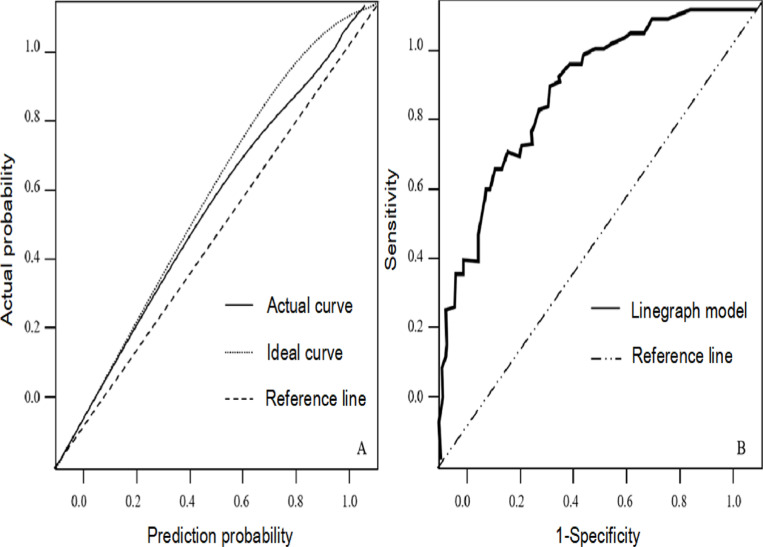
Calibration curve (A) and ROC curve (B) of nomogram model

The calibration and validity of the nomogram model were assessed. The concordance index (C-index) of the model was 0.836 (95% CI: 0.784-0.916), and the actual curve fitted well with the ideal curve in the calibration graph, indicating that the nomogram model showed good calibration in predicting the risk of post-ERCP pancreatitis in patients with gallstones (Figure 2A). The ROC curve of this nomogram model for predicting post-ERCP pancreatitis in patients with gallstones was plotted, and the model had an AUC of 0.881 (95% CI: 0.784-0.916), a specificity of 84.36% and a sensitivity of 88.61%, suggesting good discrimination (Figure 2B).

### Postoperative status of patients in surgery and non-surgery groups

Among the 193 included patients, the incidence rates of pancreatitis in patients with and without pancreatic duct stenting were 11.84% (9/76) and 39.31% (46/117), respectively (Table 4).

**Table 4:** Incidence rate of pancreatitis in patients with and without pancreatic duct stenting

Group	Pancreatitis group (n=55)	Non-pancreatitis group (n=138)
Surgery group (n=76)	9 (11.84)	67 (88.16)
Non-surgery group (n=117)	46 (39.31)	71 (60.69)
χ^2^	17.067	
P	<0.001	

In comparison with those who did not receive pancreatic duct stenting (non-surgery group), the patients undergoing pancreatic duct stenting (surgery group) had lower serum amylase levels 6, 12 and 24 h after ERCP (P<0.05) (Table 5).

**Table 5:** Serum amylase levels

Group	Serum amylase level (U/L)		

	6 h	12 h	24 h
Surgery group (n=76)	141.23±32.23	175.14±48.63	168.35±50.39
Non-surgery group (n=117)	312.52±100.26	581.36±136.57	452.91±152.35
*t*	14.407	24.906	15.722
P	0.000	0.000	0.000

## Discussion

Gallstones mostly result from poor dietary habits and specific dietary patterns^14^. At present, patients with gallstones are predominately treated by surgery. In recent years, ERCP has shown obvious therapeutic effects on gallstones, but several postoperative complications (e.g., bile duct injury, postoperative residual gallstones and pancreatitis) remain troublesome. Pancreatitis, as one of the post-ERCP complications, results in poor prognosis and unsatisfactory rehabilitation effect, probably also inducing problems such as postoperative infection^15^,^16^. After surgery for gallstones, inflammatory factors are active as a result of mechanical trauma, leading to postoperative complications^17^,^18^. Currently, the preventive effects of pancreatic duct stenting on pancreatitis have been well-documented^19^,^20^, which is characterized by mild trauma.

The mechanism of post-ERCP pancreatitis is still unclear at present. The risk factors can be categorized into patient- and operator-related factors. The patient-related factors include history of recurrent pancreatitis, sphincter of Oddi dysfunction, female, age <60 years old, cholecystectomy, periampullary diverticulum, coexisting common bile duct stones and obstructive jaundice. The operator-related factors include small papillary sphincterotomy, large pancreatic duct sphincterotomy, difficult cannulation and sphincter manometry. Bailey et al. reported that female, suspected sphincter of Oddi dysfunction and intraductal contrast filling were independent risk factors for post-ERCP pancreatitis^21^. Besides, Lee et al. reported that 13 of 200 patients (6.5%) who underwent needle-knife precut sphincterotomy suffered from post-ERCP pancreatitis, which was not significantly associated with age, incision direction, bile duct diameter or surgical success^22^.

In this study, the risk factors for post-ERCP pancreatitis in patients with gallstones were investigated and the preventive effect of pancreatic duct stenting was observed. Young age, long course of disease, gallbladder wall thickness >3 mm, sand-like stones, history of pancreatic disease and number of intubation ≥2 were verified to the risk factors for post-ERCP pancreatitis in patients with gallstones (P<0.05). Possibly, pancreatic secretion is more vigorous in younger patients with gallstones, thereby causing pancreatic damage and raising the probability of pancreatitis. Patients with a long course of disease have greater postoperative mechanical trauma, the autoimmune factors suppress the activation of digestive enzymes, and inflammatory factors are active, all of which increase the risk of pancreatitis. As for patients with gallbladder wall thickness >3 mm, the bile duct pressure is still high after operation, and high-pressure bile flows back to the pancreatic duct, causing pancreatic acini rupture and pancreatic enzyme entering the pancreatic interstitium, which increases the risk of pancreatitis. Sand-like stones mean that gallbladder atrophy is serious, so they cannot be easily removed. More residual stones after operation also increase the risk of pancreatitis. The history of pancreatic disease and the number of intubation are mostly relevant to the patient's own physical condition, and those who have poor autoimmunity, resistance and postoperative recovery are prone to pancreatitis^23^,^24^. Moreover, this study exhibited that the abdominal pain relief time, food recovery time, time of recovery to normal temperature and hospital stay were shorter in the non-pancreatitis group than those in the pancreatitis group, and the incidence rate of pancreatitis in all patients undergoing pancreatic duct stenting was 11.84% (9/76). This may be linked to the ability of this approach to rapidly improve the pancreatic duct function and to diminish the factors probably causing deterioration. However, the specific cause remains controversial at present. Andriulli et al. evaluated the preventive effects of pancreatic duct stenting on pancreatitis after ERCP examination ba meta-analysis of 6 controlled studies. Compared with the control group, the stent group had a significantly lower risk^25^.

Additionally, the nomogram model was constructed using R software. The risk value for post-ERCP pancreatitis in patients with gallstones was 0.815, i.e., the predicted probability was 81.5%, suggesting the occurrence of pancreatitis after operation. Moreover, the actual curve fitted well with the ideal curve in the calibration graph, indicating that the nomogram model had good discrimination and high accuracy.

In conclusion, patients with gallstones have a higher risk of pancreatitis. Young age, long course of disease, gallbladder wall thickness >3 mm, sand-like stones, history of pancreatic disease, pancreatic duct visualization and number of intubation ≥2 can raise the risk of pancreatitis. Therefore, targeted intervention protocols should be formulated to realize early intervention and prevention. Regardless, this study still has some limitations. First, surgical subjectivity and operative difficulties may lead to differences in the prognosis of patients. Second, the sample size was small. Thus, more clinical data are needed to determine specific therapeutic regimens such as stent selection.
